# Concurrent Impaction of the Mandibular Primary Second Molar and Second Premolar in Close Approximation to the Mental Nerve: A Case Report

**DOI:** 10.7759/cureus.57934

**Published:** 2024-04-09

**Authors:** Rajanikanth K, Nitin Bhola, Deepankar Shukla

**Affiliations:** 1 Oral and Maxillofacial Surgery, Sharad Pawar Dental College and Hospital, Wardha, IND

**Keywords:** concurrent impaction, primary 2nd premolar, primary 2nd molar, mental nerve, impacted tooth

## Abstract

Impacted teeth are those that fail to erupt at the typical age of eruption and remain enclosed in the maxilla or mandible, partially or completely surrounded by bone or soft tissues. Among these, third molars experience the highest incidence of impaction, with maxillary canines and mandibular bicuspids following closely. A 23-year-old female presented to the orthodontics department, expressing concerns about spacing issues in her upper and lower front teeth. During the orthodontic planning process and radiological assessment, two impacted teeth, specifically one primary molar and one permanent premolar, were identified in close proximity to the mental nerve. Both teeth were subsequently extracted with success. This case report underscores the importance of a thorough preoperative radiographic evaluation of the mandibular canal and foramina. Additionally, it stresses the necessity for dissection to prevent unintended injury to the mental nerve during the extraction of the impacted mandibular premolar, which can result in paresthesia affecting the lower lip, mandibular labial gingiva, and chin.

## Introduction

Clinically, around 20% of the population present with impacted teeth. Tooth impaction is characterized by a situation in which a tooth does not emerge properly because of either mechanical obstruction or inadequate space, causing it to stay within the bone beyond its expected eruption period. Pediatric dentists commonly encounter impaction issues with permanent teeth as opposed to primary ones. It is notably rare for an anterior primary tooth to be impacted. The occurrence of impacted primary teeth ranges from 1.3% to 8.9% within the population, with a notably higher frequency observed among siblings. Typically, primary mandibular molars are affected over 10 times more frequently than primary maxillary molars [1,2].

Tooth impaction is classified into primary or secondary categories. Primary impaction pertains to a tooth that has never surfaced. Secondary impaction, also recognized as a submerged primary tooth, happens when a tooth re-impacts subsequent to its initial eruption [3]. Various factors can contribute to the impaction of primary teeth, including odontomas, ankylosis, congenital absence of permanent teeth, abnormalities in the periodontal membrane, trauma, injuries to the periodontal ligament, premature eruption of the first permanent molar, deficient eruptive force, or a combination of these factors. In some cases, the exact cause is unknown and is thought to have a genetic basis. On the other hand, secondary impaction typically occurs due to a gradual decrease in occlusal contact without a corresponding increase in the height of the alveolar process beneath the submerged deciduous tooth. Conversely, the adjacent alveolar processes shift occlusally because of the emergence of neighboring permanent teeth, leading to the complete embedding of the primary tooth in oral tissues and positioning it below the occlusal plane. This present article describes a case report on the extraction of an impacted mandibular primary second molar and second premolar in close approximation to the mental nerve.

## Case presentation

A 23-year-old female patient reported to the Department of Orthodontics, Sharad Pawar Dental College & Hospital, Datta Meghe Institute of Higher Education & Research, Sawangi (M), Wardha with a chief complaint of spacing in anteriors and wanted to get it corrected. Orthodontic treatment was planned for space closure. During the planning of orthodontic treatment, different radiographs were taken, like orthopantomogram (OPG) and cone-beam computed tomography (CBCT), which revealed an impacted primary second molar and second premolar. The patient was referred to the Department of Oral and Maxillofacial Surgery for the surgical extraction of the impacted tooth.

**Figure 1:**
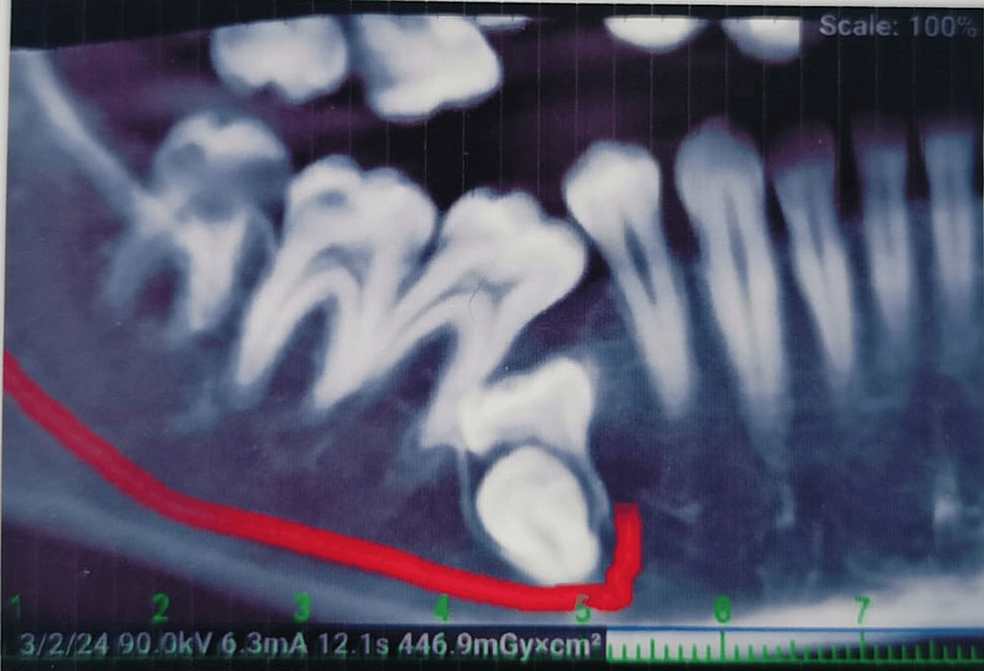
Radiograph of the impacted site

**Figure 2:**
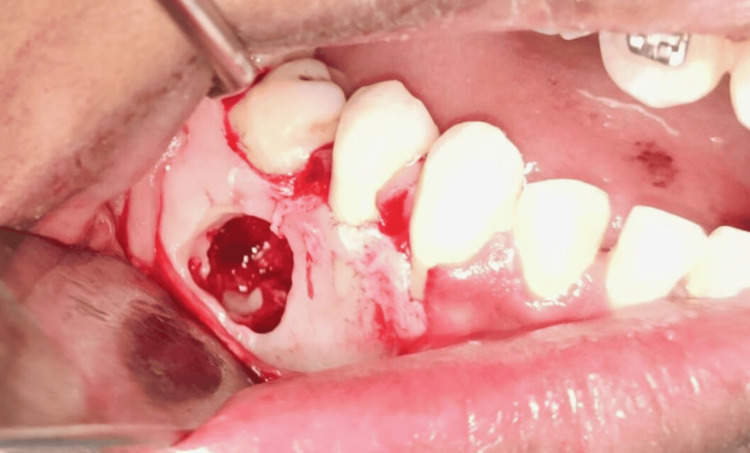
Bony window showing the impacted deciduous tooth

**Figure 3:**
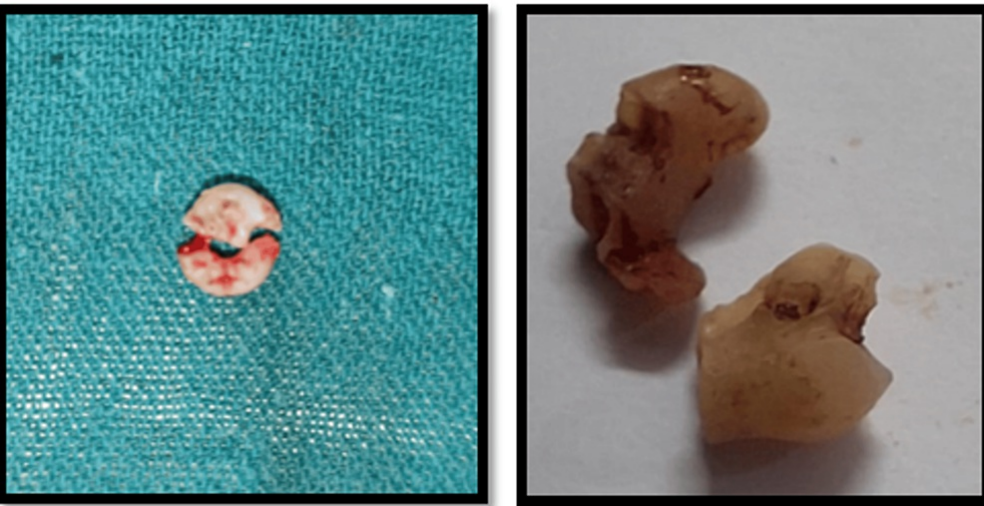
Sectioned fragments of the impacted deciduous molar and premolar teeth

After the clinical and radiographic evaluations (Figure 1), and having evaluated the various possible treatment options, in this present case, it was planned to perform surgery for the extraction of the impacted deciduous tooth and permanent tooth. Local anesthesia lignocaine 2% with 1:100000 adrenaline was administered and a sulcular incision was given to elevate a trapezoidal flap (full-thickness mucoperiosteal flap), taking care of the mental nerve. A bony window (Figure 2) was created using a rotary tool micromotor with carbide bur under saline irrigation and the deciduous molar was exposed and sectioned. Through the same access, sectioning of the impacted premolar was done near the cementoenamel junction, and the tooth was removed in fragments (Figure 3). Subsequently, the flap was repositioned and sutured using non-absorbable sutures (3-0 silk). The patient was kept under follow-up. There was mild paraesthesia on the lower lip, which resolved in 10 days.

## Discussion

Options for managing impacted teeth encompass observation, intervention, relocation, and extraction. Occasionally, these treatment modalities may interact with one another [4,5]. The shedding of deciduous teeth is a typical and expected physiological process [6]. The specific mechanism of exfoliation in primary teeth involves the resorption of the primary root under pressure, stimulated by the emerging successional tooth. Another aspect of this process involves the transformation of monocytes within the periodontal ligament into odontoclasts. These odontoclasts then dissolve the primary root, resembling the action of osteoclasts in bone remodeling or resorption, all without inducing an inflammatory reaction. The factors that initiate this process are not yet fully understood. It is noteworthy that the majority of decayed primary teeth undergo natural exfoliation and do not necessarily require restoration [7,8].

In the current scenario, an impacted second premolar and second molar were detected during the ongoing orthodontic procedure, and this was subsequently confirmed through radiographic assessment. A panoramic radiograph was employed for radiographic assessment, revealing an infraoccluded mandibular right second primary molar with an emerging mandibular right second premolar tooth germ. Further examination using three-dimensional CBCT displayed an impacted second primary molar and an associated displaced permanent successor tooth germ situated beneath the resorbed roots of the second primary molar [9]. Very few cases are reported in the literature with concurrent impaction of deciduous molar and associated premolar. Timely identification may have helped in orthodontic alignment of the premolar, but as the patient reported late, a decision of surgical removal of both teeth was taken.

Abundant evidence in the literature indicates that genes significantly contribute to the development of various clinically significant dental anomalies. Consequently, a shared genetic abnormality may lead to diverse phenotypic expressions, such as tooth agenesis, delayed development, and ectopic eruption [10]. Antoniades et al. noted that primary tooth impaction occurs twice as often in the mandible compared to the maxilla, with mandibular second molars being the most frequently affected. Consistent with this, the present case involved an impacted mandibular primary second molar [11].

Accurate diagnosis of primary tooth impaction relies on specific criteria, encompassing deep embedding within the bone, the absence of decay or dental restorations on the crown, a lack of root resorption, regular emergence of the corresponding permanent tooth, and potential retention and misalignment of the adjacent permanent tooth. Frequently associated with the impaction of primary teeth is the absence of their permanent successors, often stemming from agenesis. Prior to initiating any treatment, a crucial consideration involves evaluating the potential for self-correction of the deviation from the normal eruption path. Should spontaneous realignment not occur following a brief observation period, active treatment becomes a prudent course of action. The typical treatment approach for an impacted primary tooth involves enucleation, although this procedure carries potential risks such as damage to the mental foramen or inferior dental canal. Studies indicate that the position of the mental foramen can vary; for instance, Fishel et al. revealed that the mental foramen is situated between the apices of two premolars in approximately 70.4% of cases. However, other studies suggest the existence of vertical variations in the position of the mental foramen [12]. Therefore, it is crucial to establish the positions of the inferior dental canal and mental foramen in relation to the impacted tooth, and careful evaluation of the inferior alveolar nerve bundle and mental nerve is necessary before surgery. In the described case, the treatment plan involved a radical surgical intervention with the extraction of the impacted tooth. During the procedure, the mental nerve was identified and delicately dissected from surrounding tissue to allow for some laxity during tissue handling and minimize the risk of injury, resulting in an improved postoperative outcome [13-16].

## Conclusions

Detecting and promptly extracting impacted teeth, which can result in the formation of odontomas and cystic lesions, holds the potential for better outcomes in achieving optimal occlusion and functionality. Furthermore, the judicious utilization of CBCT is strongly advised in specific scenarios. Dentists should consider opting for CBCT imaging when they anticipate substantial improvements in patient care, safety, or clinical outcomes. This technology provides dental professionals with a high-resolution, minimally distorted, and low-radiation imaging tool, offering a detailed three-dimensional representation of the maxillofacial skeleton.
